# Neuropathological Responses to Chronic NMDA in Rats Are Worsened by Dietary n-3 PUFA Deprivation but Are Not Ameliorated by Fish Oil Supplementation

**DOI:** 10.1371/journal.pone.0095318

**Published:** 2014-05-05

**Authors:** Vasken L. Keleshian, Matthew Kellom, Hyung-Wook Kim, Ameer Y. Taha, Yewon Cheon, Miki Igarashi, Stanley I. Rapoport, Jagadeesh S. Rao

**Affiliations:** 1 Virginia Commonwealth University, School of Medicine, Richmond, Virginia, United States of America; 2 School of Earth and Space Exploration, Arizona State University, Phoenix, Arizona, United States of America; 3 College of Life Sciences, Sejong University, Gunja-dong, Gwangjin-Gu, Seoul, Korea; 4 Brain Physiology and Metabolism Section, Laboratory of Neurosciences, National Institute on Aging, NIH, Bethesda, Maryland, United States of America; 5 Department of Anatomy & Neurobiology, School of Medicine, University of California Irvine, Irvine, California, United States of America; Virginia Tech, United States of America

## Abstract

**Background:**

Dietary long-chain n-3 polyunsaturated fatty acid (PUFA) supplementation may be beneficial for chronic brain illnesses, but the issue is not agreed on. We examined effects of dietary n-3 PUFA deprivation or supplementation, compared with an n-3 PUFA adequate diet (containing alpha-linolenic acid [18:3 n-3] but not docosahexaenoic acid [DHA, 22:6n-3]), on brain markers of lipid metabolism and excitotoxicity, in rats treated chronically with NMDA or saline.

**Methods:**

Male rats after weaning were maintained on one of three diets for 15 weeks. After 12 weeks, each diet group was injected i.p. daily with saline (1 ml/kg) or a subconvulsive dose of NMDA (25 mg/kg) for 3 additional weeks. Then, brain fatty acid concentrations and various markers of excitotoxicity and fatty acid metabolism were measured.

**Results:**

Compared to the diet-adequate group, brain DHA concentration was reduced, while n-6 docosapentaenoic acid (DPA, 22:5n-6) concentration was increased in the n-3 deficient group; arachidonic acid (AA, 20:4n-6) concentration was unchanged. These concentrations were unaffected by fish oil supplementation. Chronic NMDA increased brain cPLA_2_ activity in each of the three groups, but n-3 PUFA deprivation or fish oil did not change cPLA_2_ activity or protein compared with the adequate group. sPLA_2_ expression was unchanged in the three conditions, whereas iPLA_2_ expression was reduced by deprivation but not changed by supplementation. BDNF protein was reduced by NMDA in N-3 PUFA deficient rats, but protein levels of IL-1β, NGF, and GFAP did not differ between groups.

**Conclusions:**

N-3 PUFA deprivation significantly worsened several pathological NMDA-induced changes produced in diet adequate rats, whereas n-3 PUFA supplementation did not affect NMDA induced changes. Supplementation may not be critical for this measured neuropathology once the diet has an adequate n-3 PUFA content.

## Introduction

The central nervous system is highly enriched in the polyunsaturated fatty acids (PUFAs) arachidonic acid (AA, 20:4n-6) and docosahexaenoic acid (DHA, 22:6n-3). AA and DHA cannot be synthesized *de novo* by vertebrates, but must be obtained from the diet or by hepatic elongation and desaturation of their dietary essential precursors, linoleic acid (LA, 18:2n-6) and α-linolenic acid (α-LNA, 18:3n-3), respectively [1], [2]. The balance between AA and DHA is crucial for normal brain function [3], [4]. AA is a precursor of prostaglandins, thromboxanes, leukotrienes, and related compounds that have important roles in inflammation and in the regulation of brain immunity. DHA and its metabolites are thought to be neuroprotective and to have anti-inflammatory effects [5]. AA signaling in brain is mediated by Ca^2+^-dependent cytosolic phospholipase A_2_ (cPLA_2_), while DHA signaling is mediated by Ca^2+^-independent phospholipase A_2_ (iPLA_2_) [6].

North American diets tend to contain low levels of DHA and high levels of n-6 PUFAs derived primarily from LA [7]–[9]. Some epidemiological studies suggest that reduced dietary n-3 PUFA content is associated with an increased risk of neuropsychiatric and neurodegenerative disorders [10], in which excitotoxicity is present [11], [12].

Animal studies have been performed to understand how diet-derived n-3 PUFAs influence body integrity and metabolism. In rats, a DHA-free diet containing α-LNA at 4.6% of total fatty acid is sufficient to maintain normal growth and organ function, so this diet is considered n-3 PUFA “adequate” [1], [13]. In contrast, in rats fed a DHA-free diet containing low amounts of α-LNA (0.2% of dietary fatty acids), brain DHA concentration is reduced, behavior is disturbed and brain derived neurotrophic factor (BDNF) is reduced compared with the adequate 4.6% α-LNA diet, so this diet is considered n-3 PUFA “inadequate” or “deficient” [13]–[15]. Brain changes in rats fed the n-3 PUFA deficient diet include a prolonged DHA half-life, an increased concentration of docosapentaenoic acid (DPAn-6, 22:5n-6) derived from liver elongation of AA, and reduced expression of DHA-metabolizing iPLA_2_ [16], [17] and cyclooxygenase (COX)-1 enzymes [17]. Additionally, n-3 PUFA deprivation increases brain expression of enzymes that regulate AA metabolism, including cPLA_2_ Type IVA, sPLA_2_ Type II and COX-2 [17].

Effects of n-3 PUFA deprivation and supplementation on brain markers during physiological or pharmacological insults are uncertain. Several studies reported that dietary n-3 PUFA deficiency affected brain glutamatergic, dopaminergic and serotonergic systems [16]–[18]. Hyperglutamatergic activity is reported in neurodegenerative and neuropsychiatric illnesses such as Alzheimer disease (AD) [11], Parkinson disease [19] and bipolar disorder [20], [21]. Glutamate is the main excitatory neurotransmitter in brain and is involved in learning and memory, brain development and aging, and excitotoxicity [21]–[23]. Glutamate exerts some of its effects *via* ionotropic *N*-methyl-D-aspartate [NMDA], alpha-amino-3-hydroxy-5-methyl-4-isoxazolepropionate [AMPA] and kainate receptors, and *via* G-protein coupled metabotropic glutamate receptors [24]. We reported that a chronic daily subconvulsive dose of NMDA (25 mg/kg i.p.) in adult rats decreased brain NMDA receptor subunit levels [21], and upregulated mRNA and protein levels of interleukin-1 beta (IL-1β), tumor necrosis factor alpha (TNF-α), glial fibrillary acidic protein (GFAP) and inducible nitric oxide synthase (iNOS) in the frontal cortex [25]. Furthermore, chronic NMDA increased AA turnover in rat brain phospholipids, and upregulated cPLA_2_ IVA mRNA, protein and activity, and the cPLA_2_ transcription factor activation protein AP-2 [21].

The extent of dietary n-3 PUFA protection against excitotoxicity is not agreed on. We designed this study to understand whether dietary n-3 PUFA deficiency or supplementation could modify chronic NMDA-induced changes in rat brain AA signaling, neuroinflammatory markers and trophic factors. To do this, we maintained rats on an n-3 PUFA deprived, adequate or supplemented (by fish oil) diet for 15 weeks after weaning. After the initial 12 weeks, each of the three groups of animals was administered saline or a subconvulsive dose of NMDA (25 mg/kg, i.p.) daily for 3 weeks. The rats were sacrificed at 15 weeks and their brains were used to measure lipid concentrations, and molecular markers of lipid metabolism, neuroinflammation, BDNF and nerve growth factor (NGF).

## Methods

### Ethics Statement

This protocol was approved by the Animal Care and Use Committee of the *Eunice Kennedy Shriver* National Institute of Child Health and Human Development, and followed the National Institutes of Health Guide for the Care and Use of Laboratory Animals (NIH Publication 86–23).

### Animals

Eighteen to 20 day-old male CDF rat pups weighing 30–40 g, and their nursing surrogate mothers, were purchased from Charles River Laboratories (Portage, MI, USA). The pups were not littermates. The animals were housed in a facility with regulated temperature (24°C) and humidity (40–70%), under a 12 h light/dark cycle. They were allowed to nurse until 21 days old, then removed from their mothers, mixed without identification, and assigned randomly to one of three dietary groups: an n-3 PUFA adequate diet (4.6% α-LNA of total fatty acids, no DHA), fish oil supplemented diet (5.1% α-LNA of total fatty acids, 1.9% EPA and 2.4% DHA) (Zeigler Bros, Gardners, PA, USA) and an n-3 PUFA deficient diet (α-LNA is 0.2% of total fatty acids, no DHA) (Dyets, Inc. Bethlehem, PA, USA). The specific diet compositions are reported elsewhere [26], [27].

The n-3 PUFA and deficient diets were based on the AIN-93 diet and differed with regard to fat composition. The n-3 PUFA adequate diet contained 6.0, 3.2 and 0.8 (g/100 g diet) hydrogenated coconut oil, safflower oil and flaxseed oil, respectively, while the n-3 PUFA deficient diet contained 6.6, 3.4 and 0 (g/100 g diet) hydrogenated coconut oil, safflower oil and flaxseed oil, respectively. The fish oil supplemented diet was based on the NIH-31 diet which contains EPA and DHA. The composition of the diets is provided in Table 1. We used these three diets in particular because we previously reported their effects on PUFA metabolism in the brain and liver of rats. There is no difference in brain DHA quantity, DHA turnover, and brain DHA incorporation between rats fed the NIH 31 fish oil diet and the n-3 PUFA adequate DHA-free diet even when dietary composition is not matched [28], [29].

**Table 1:** Dietary compositions of n-3 PUFA adequate, n-3 PUFA deficient and NIH-31 diets.

	n-3 PUFA adequate diet^1^	n-3 PUFA deficient diet^1^	NIH -31^2^
	g/100 g diet	g/100 g diet	g/100 g diet
Protein	20	20	18
Carbohydrate	60	60	65
Fat	10	10	4.7
Ash	1	1	8
Fatty acids	% of total fatty acids	% of total fatty acids	% of total fatty acids
14:0	12.5	14.60	1.4
14:1 n-5	0.04	0.05	N/A
16.0	9.5	9.9	15.7
16:1n-7	0.04	0.13	1.5
18:0	8.0	8.0	3.2
18:1 n-9	8.4	5.9	20.60
18:2 n-6	27.9	27.20	47.5
18:3 n-3	4.6	0.20	5.1
20:5n-3 (EPA)	None	None	1.9
22:6-n-3 (DHA)	None	None	2.4
Total Saturated	58.8	66.50	0.90
Total monosaturated	8.5	6.1	1.20
Total n-6 PUFA	27.9	27.20	18.8
Total n-3 PUFA	4.6	0.2	9.4
n-6/n-3	6.1	136	2
Fatty acid content µmol/g diet			
18:2 n-6	40.5	35.8	59.0
18:3 n-3	6.7	0.21	5.9

1 Adapted from reference [27].

2 Composition of the NIH-31 diet was determined by gas-chromatography.

After 12 weeks on a dietary regimen, rats in each group (n = 16 per group) received vehicle (0.9% saline, n = 8) or NMDA (25 mg/kg body weight, n = 8) (Sigma Aldrich, St. Louis, MO, USA) by intraperitoneal injection once daily for 21 days. The rats were maintained on their respective diets for three more weeks. Three hours after the last saline or NMDA injection, a rat was anesthetized with an overdose of CO_2_ and decapitated. The brain was rapidly excised and cut sagittally into four sections, which were used for lipid analysis, Western blotting, mRNA analysis and enzyme activity assays. Brain sections were frozen in 2-methylbutane, and stored at −80°C until further use.

During the course of NMDA treatment, one rat from each of the n-3 deficient and n-3 adequate groups treated with NMDA was euthanized because they exhibited a spontaneous tonic-clonic seizure. The lipid and molecular biology analyses were done on 6 out of the remaining 7 or 8 animals per group.

### Brain lipid composition analysis

Brain total lipids were extracted with chloroform/methanol (2∶1 v/v) from one quarter of brain, derivitized in 1% methanolic H_2_SO_4_ and analyzed by gas chromatography as previously described [30].

### Preparation of cytosolic fractions for Western blot analysis

Cytosolic brain fractions were prepared as reported [31]. Briefly, one quarter section of the left hemisphere was homogenized in a buffer containing 20 mM Tris-HCl (pH 7.4), 2 mM EGTA, 5 mM EDTA, 1.5 mM pepstatin, 2 mM leupeptin, 0.5 mM phenylmethyl sulfonylfluoride, 0.2 U/ml aprotinin, and 2 mM dithiothreitol, using a Polytron homogenizer. The homogenate was centrifuged at 100,000 g for 60 min at 4°C, and the resulting supernatant (cytosolic fraction) was collected. Protein concentration of the cytosolic fraction was determined using Bio-Rad Protein Reagent (Bio-Rad, Hercules, CA, USA).

### PLA_2_ enzyme activities

Activities of individual PLA_2_ enzymes were measured in the cytosolic fraction as described in detail elsewhere [11], [32], with slight modifications to the extraction. For cPLA_2_ activity, a portion of the cytosolic fraction was incubated in 100 mol/L 1-palmitoyl-2-arachidonoyl-sn-glycerol-3-phosphorylcholine (Avanti, Alabaster, AL, USA) and phosphatidylinositol 4,5-bisphosphate (97∶3) containing approximately 100.000 cpm of 1-palmitoyl-2-[1-^14^C] arachidonoyl-sn-glycerol-3-phosphorylcholine (Perkin-Elmer, Boston, MA, USA) and 4,5 biphosphatidylinositol (Avanti) in 400 mol/L triton X-100 mixed micelles containing 100 mmol/L Hepes, pH 7.5, 80 mol/L calcium, 2 mmol/L DTT, and 0.1 mg/mL fatty acid free bovine serum albumin. For iPLA_2_ activity, a portion of the cytosolic fraction was incubated in 100 µmol/L 1-palmitoyl-2-palmitoyl-sn-glycerol-3-phosphorylcholine (Avanti) containing approximately 100,000 cpm of 1-palmitoyl-2-[1-^14^C] palmitoyl-sn-glycerol-3-phosphorylcholine (Amersham, Buckinghamshire, UK) in 400 µmol/L Triton X-mixed micelles in 100 mmol/L HEPES, pH 7.5, 5 mmol/L EDTA, 2 mmol/L DTT, and 1 mmol/L ATP.

Assays were started by adding reagent to cytoplasmic extracts (0.3 mg in one assay) for 30 min at 40°C in a shaking bath. Reactions were terminated by adding Dole's reagent (2-propanol: heptane: 0.5 mol/L sulfuric acid, 400: 100: 20, by volume) followed by vortexing. Released [1-^14^C] fatty acids were extracted with the addition of heptane and water. One milliliter of the heptane was loaded on a bond elute reservoir with a frit pre-loaded with silicic acid. The unesterified [1-^14^C] fatty acids were eluted from the silicic acid by adding diethyl ether with the help of a vacuum. Radioactivity of the eluant was determined by liquid scintillation counting and activity was calculated after correcting for the background of blank samples. All samples were run in triplicate and values are expressed in pmol/min/mg of protein.

#### sPLA_2_ activity

sPLA_2_ activity was measured using an appropriate assay kit (Cayman Chemical, Ann Arbor, MI), according to the manufacturer's instructions.

### Western blot analysis of protein levels

Proteins from the cytosolic fraction (65 µg) were separated on 4–20% SDS-polyacrylamide gels (PAGE) (Bio-Rad), and electrophoretically transferred to a nitrocellulose membrane (Bio-Rad). Cytosolic protein blots were incubated overnight in Tris-buffered-saline containing 5% nonfat dried milk and 0.1% Tween-20, with specific primary antibodies for proinflammatory cytokines: IL-1β (1∶500), GFAP (1∶1000), cPLA_2_-IVA, sPLA_2_-IIA, iPLA_2_-VIA, COX-1 (1∶500) and COX-2 (1∶200) (Santa Cruz Biotechnology, Santa Cruz, CA, USA); and β-actin (1∶10,000) as a housekeeping protein (Sigma Aldrich). The cytosolic blots were incubated with appropriate horseradish peroxidase (HRP)-conjugated secondary antibodies (Bio-Rad), and were visualized using a chemiluminescence reaction (Amersham, Piscataway, NJ, USA). Optical densities of immunoblot bands were measured using Alpha Innotech Software (Alpha Innotech, San Leandro, CA, USA) and were normalized to β-actin. All experiments were conducted on six independent samples. Mean values are expressed as percent of control.

### BDNF and NGF protein determination

BDNF and NGF protein levels were determined with respective ELISA kits (Sigma Aldrich) following the manufacturer's recommendations. Values are expressed as percent of control.

### Statistics

A two-way ANOVA was used to determine main effects of diet and NMDA on brain fatty acid concentrations and activity, mRNA and protein levels. A one-way ANOVA followed by Newman-Keuls Multiple Comparison post-hoc test was used to compare the differences between the adequate-saline vs. n-3 PUFA deprived saline or fish oil-saline groups. Statistical significance also was calculated among the three groups (n-3 PUFA adequate saline vs. n-3 PUFA adequate –NMDA, n-3 PUFA deprived vs. n—3 PUFA –NMDA group and Fish oil-Saline vs. Fish oil-NMDA group). Data are expressed as mean ± SEM. Statistical significance is denoted by * p≤0.05, ** p≤0.01, *** p≤0.001.

## Results

**Figure 1:**
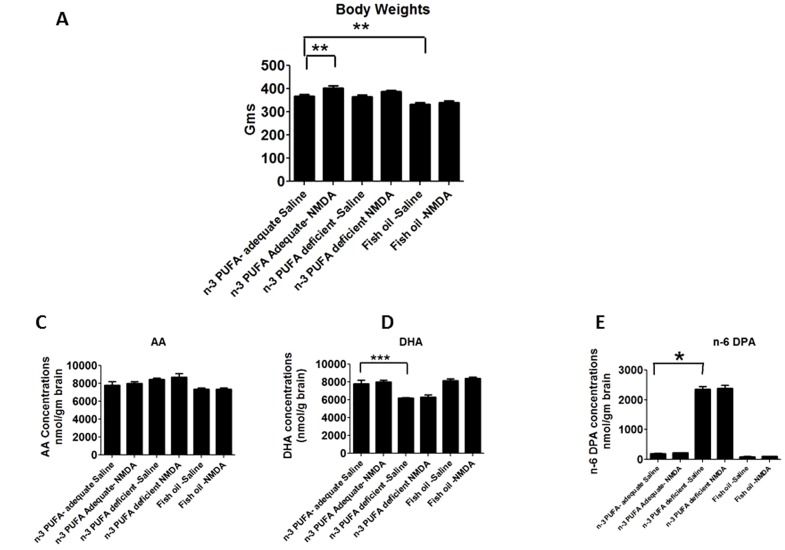
Brain lipid composition and activity of AA cascade enzymes. Brain lipid composition in nmol/g brain of A) Body weights B) AA C) DHA D) n-6 DPA. Data are expressed as mean ± SEM (n = 6 per group). Statistical significance is denoted by * p≤0.05, ** p<0.01, *** p<0.001.

There was a significant main NMDA and diet effect, and NMDA and diet interaction on body weight (p<0.05 by two-way ANOVA). At 15 weeks, mean body weight of fish oil-saline administered rats was significantly less compared to weight of the adequate group-saline administered rats (Fig. 1A). However, body weight was significantly increased in diet-adequate rats given chronic NMDA compared with saline (Fig. 1A).

### Brain lipid composition

A two-way ANOVA showed no significant main effect of NMDA or interaction of diet x NMDA on brain AA, DPA n-6 and DHA concentrations with all the three n-3 PUFA diets. There was a significant diet effect on PUFA concentrations with each of the three diets (Fig. 1). The brain AA, DPA n-6 and DHA concentrations did not differ significantly between NMDA and saline administered rats in each diet group (Fig. 1B). DPAn-6 concentration was significantly higher, and DHA concentration was significantly lower, in saline treated rats from the n-3 PUFA deprived compared to adequate group (Figs. 1C and 1D). Fish oil supplementation did not alter brain AA, DHA or n-6 DPA concentration compared to respective concentrations with the adequate diet (Figs. 1B, C, and D).

### Effect of diet and NMDA on arachidonic acid cascade markers

**Figure 2:**
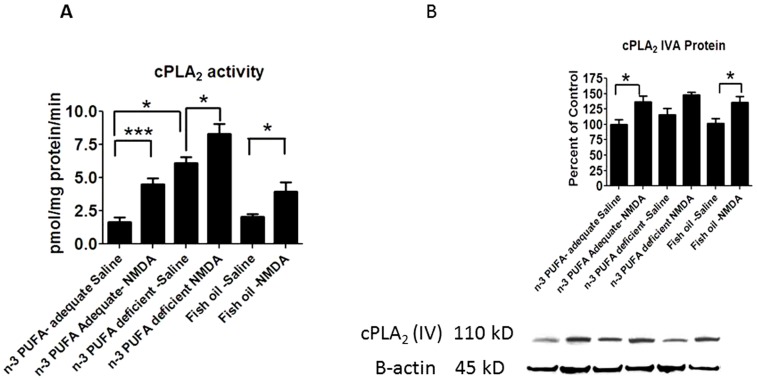
cPLA_2_ activity and protein expression. Measured activity of A) cPLA_2_ enzymes and B) cPLA_2_ protein levels. Data are expressed as mean ± SEM (n = 6 per group for activity, or n = 5-6 per group for protein, due to unquantifiable bands). Statistical significance is denoted by * p≤0.05, ** p<0.01, *** p<0.001.

A two-way ANOVA showed a significant diet and NMDA main effect on brain cPLA_2_ activity. Chronic NMDA compared with saline significantly increased cPLA_2_ activity in each of the three dietary groups (Fig. 2A). cPLA_2_ activity with chronic saline was significantly increased in n-3 PUFA deprived compared with the adequate group (3.4 fold: p<0.0001), but unchanged by fish oil supplementation (Fig. 2A). The same two-way ANOVA analysis showed a significant NMDA effect but no significant interaction of diet x NMDA on brain cPLA_2_ protein with the three diets. Whereas diet had no effect on cPLA_2_ protein in saline treated rats, chronic NMDA compared with saline significantly elevated cPLA_2_ IVA protein in rats fed the n-3 adequate or supplemented diet but not the deprived diet (Fig. 2B).

**Figure 3:**
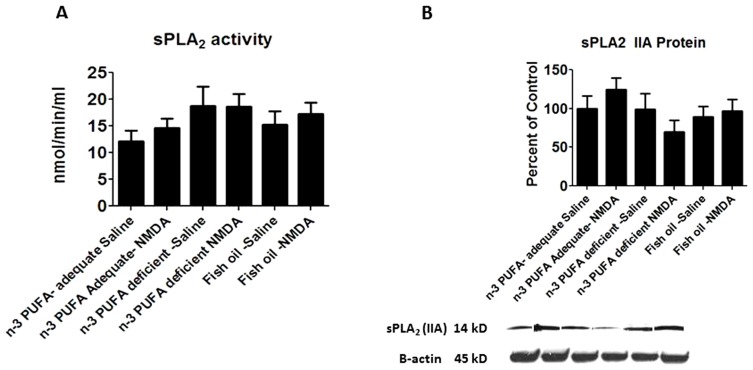
sPLA_2_ activity and protein expression. Measured activity of A) sPLA_2_ enzymes and B) sPLA_2_ protein levels. Data are expressed as mean ± SEM (n = 5–6 per group for activity due to a negative activity value of −2.37 in the fish oil-NMDA group that was removed, and 5 per group for protein due to unquantifiable bands). Statistical significance is denoted by * p≤0.05, ** p<0.01, *** p<0.001.

Mean sPLA_2_ activity and protein levels were not significantly changed (Figs. 3A and 3B), and a two-way ANOVA showed no significant diet x NMDA interaction, or main effects on sPLA_2_ IIA protein.

**Figure 4:**
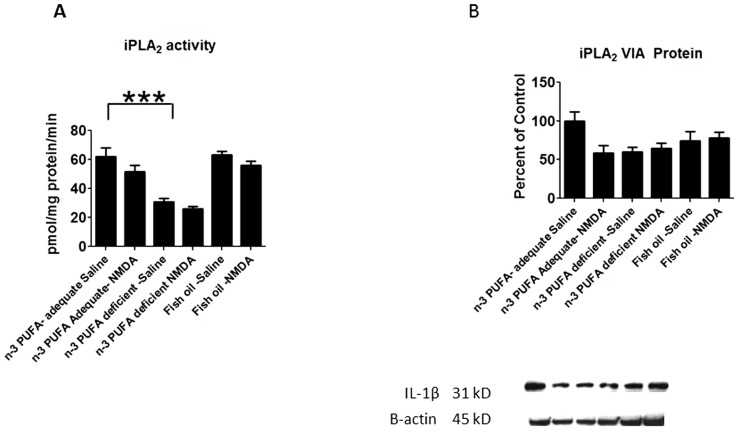
iPLA_2_ activity and protein expression. Measured activity of A) iPLA_2_ enzymes and B) iPLA_2_ protein levels. Data are expressed as mean ± SEM (n = 6 per group for activity, and 5 per group for protein due to one unquantifiable band per group). Statistical significance is denoted by * p≤0.05, ** p<0.01, *** p<0.001.

A two-way ANOVA showed a significant diet (P = 0.0013) and NMDA (p = 0.0043) effect on iPLA_2_ activity. Mean iPLA_2_ activity was decreased significantly in the n-3 PUFA deprived compared to adequate rats given chronic saline (Fig. 4A), but iPLA_2_ activity was not increased by supplementation. A two-way ANOVA showed a significant diet x NMDA interaction (p = 0.0376) on iPLA_2_ VIA protein (Fig. 4B). Post-hoc analysis with one-way ANOVA showed no statistically significant differences between the groups (p = 0.054).

**Figure 5:**
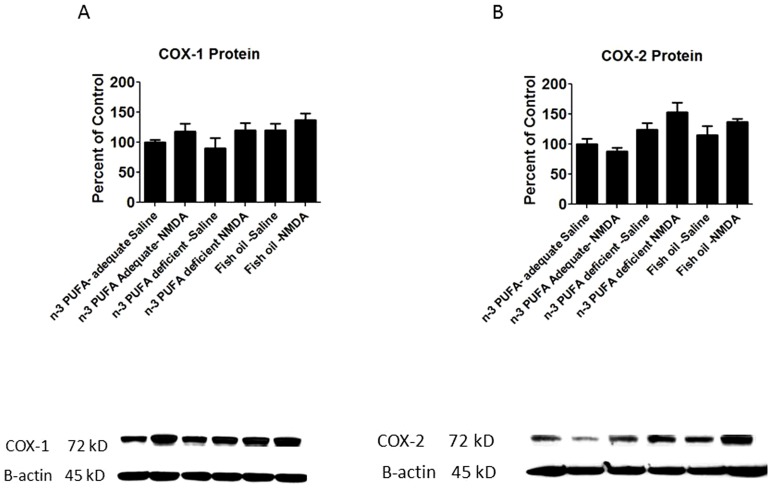
COX protein level. Measured protein levels of A) COX-1 and B) COX-2. Data are expressed as mean ± SEM (n = 5 per group for protein due to one unquantifiable band per group). Statistical significance is denoted by * p≤0.05, ** p<0.01, *** p<0.001.

There was a main effect of diet (p = 0.0016) and NMDA (p = 0.039) on COX-2 and COX-1 protein. However, post-hoc analysis by one-way ANOVA showed that chronic NMDA compared with chronic saline did not significantly alter the COX-1 or COX-2 protein level in the deficient or supplemented compared with adequate diet group (Figs. 5A and B), and in each of the three groups NMDA did not have a significant effect on protein levels.

### Effects of diet on neuroinflammatory markers and neurotrophic factors

**Figure 6:**
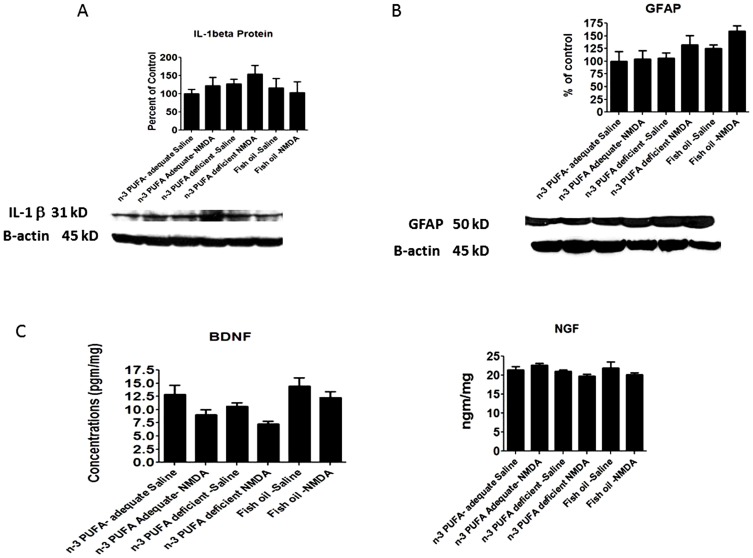
Neuroinflammatory cytokine and neurotrophin gene expression. Mean protein levels of A) IL-1β, B) GFAP C)BDNF D) NGF. Data are expressed as mean ± SEM (n = 4–5 per group for IL-1β, 4–6 per group for GFAP, 5–6 per group for BDNF, and 6 per group for NGF. Some groups had less than n = 6 due to unquantifiable bands). Statistical significance is denoted by * p≤0.05, ** p<0.01, *** p<0.001.

Protein levels of the astrocytic marker GFAP and of inflammatory cytokine IL-1b were not significantly changed by diet in the saline treated rats (Figs. 6A and B). There was a significant main effect of diet (p = 0.0033) and NMDA (0.0032) on BDNF protein, although post-hoc analysis by one-way ANOVA did not show significant differences in the deficient or fish oil groups compared to the adequate group, or between saline and NMDA treatment within each diet (Fig. 4C). NGF protein was not significantly altered by diet or NMDA in all the dietary groups (Fig. 4D).

## Discussion

This study confirms that chronic NMDA administration compared with saline control produces a number of changes in brain lipid metabolism, markers of neuroinflammation and growth factors in n-3 PUFA diet adequate rats. Dietary n-3 PUFA deprivation below the adequate level exacerbated these changes, whereas supplementation did not dampen them. In n-3 PUFA adequate rats fed 4.6% a-LNA but no DHA, NMDA compared with saline increased body weight, cPLA_2_ activity and cPLA_2_ IVA protein, but decreased iPLA_2_ protein and BDNF. Deprivation to an α-LNA content of 0.2% of total fatty acid, significantly changed NMDA responses compared with saline treated group and increase cPLA_2_ activity and protein was observed. In rats supplemented with n-3 PUFA via fish oil, NMDA compared with saline also increased cPLA_2_ activity and cPLA_2_ IVA protein.

Significant diet effects as noted by ANOVAs and post-hoc comparisons with the adequate group occurred in DHA and DPAn-6 concentrations (DHA decreased and DPAn-6 increased with n-3 PUFA deprivation), body weight (decreased by supplementation), cPLA_2_ activity (increased by deprivation) and iPLA_2_ activity (decreased by deprivation). Thus, deprivation worsened or did not change pathological markers induced by chronic NMDA in rats fed the adequate diet, whereas supplementation did not affect these markers significantly but did reduce body weight. In agreement with our findings, dietary n-3 PUFA supplementation reduced weight gain and improved postprandial lipidemia and the associated inflammatory response in the obese JCR:LA-cp rat [33], whereas dietary n-3 PUFA deprivation did not change body weight [34].

Clinical reports indicate that dietary supplementation of long-chain n-3 PUFAs can correct some behavioral or neurocognitive symptoms in bipolar disorder and Alzheimer disease patients, and that low n-3 PUFA intake may exacerbate these symptoms [35]–[38]. Rodent studies suggest that reduced dietary n-3 PUFA increases brain pro-inflammatory markers such as AA cascade enzymes and reduces the neurotrophic factor BDNF [21], [39]. In the present study, we found that 15 weeks of n-3 PUFA deprivation increased NMDA-induced brain cPLA_2_ activity and protein levels. Deprivation also reduced iPLA_2_ activity and protein as well as BDNF protein. Deprivation increased the brain concentration of n-6 DPA while reducing DHA concentration, whereas supplementation had no significant effect on any of the three concentrations. Although, supplementation may not provide significant protection against NMDA excitotoxicity if the adequate dietary requirement of n-3 PUFA is satisfied, supplementation might be helpful where liver damage or genetic alterations in n-3 PUFA metabolic enzymes exist.

The dietary ratio of AA to DHA affects various brain signaling pathways including protein kinases A and C and MAPK p38 [21]. Changes in these protein kinases were reported to affect BDNF in rat brain [40]. The current study supports previous findings showing decline in BDNF with deprivation. BDNF knockdown/knockout mice have increased depressive behavioral scores [40]. Increased AA metabolism has been linked to apoptosis with loss of BDNF [41]. These findings suggest that an increase in AA metabolism due to increased cPLA_2_ activity after n-3 PUFA deprivation and/or an NMDA insult can affect neuronal integrity, plasticity and behavior.

Chronic NMDA independently promotes neuronal death and pro- and anti-apoptotic factors in rat brain [42]. An association between increased expression of AA cascade enzymes and neurocognitive impairments/neurodegeneration has been suggested for Alzheimer disease and vascular dementia [43], [45]. The present study suggests that n-3 PUFA deprivation upregulates AA metabolism and amplifies the NMDA insult. These changes could contribute to cognitive impairment, while attenuation of AA release by inhibiting cPLA_2_ may be beneficial. Future studies might examine effects of dietary supplementation in the presence and absence of an NMDA antagonist on chronic NMDA induced changes in rat brain.

Our findings may clarify whether dietary n-3 PUFA supplementation is necessary in human subjects [45]. The brain DHA concentration did not significantly differ between adequate vs. fish oil supplemented diets and it seems that slight reductions in circulating DHA in humans would not necessarily have pathophysiological consequences. Supporting this suggestion, a 33% lesser blood DHA concentration in vegetarians than in omnivores [46] was not associated with a significant difference in mood, or in general mortality or mortality from any cause [47], [48]. Moreover, 4-month dietary DHA supplementation in preschool children, which increased blood DHA from 1.0% to 3.2% of total fatty acids, did not enhance scores on any of four cognitive tests [49].

There are two limitations with this study. First, the fat composition of the n-3 PUFA supplemented (fish oil) diet was 50% lower than that of the n-3 adequate and deficient diets (Table 1). This may explain the lower body weights of the supplemented group compared to the adequate and deficient groups. Second, the LA composition of the fish oil diet was 1.75 times higher than that of the adequate and deficient groups. This may explain why the fish oil supplemented diet did not prevent NMDA-induced changes in AA cascade and neuroinflammatory markers. In agreement with this suggestion, one study reported a reduction in headache frequency in chronic migraine patients, in which peripheral AA metabolism is upregulated, when fish oil supplementation was combined with a low LA diet [50]. Fish oil supplementation may be clinically beneficial when LA is substantially reduced. The possible benefits of combining fish oil with a low LA diet should be explored in future studies.

In conclusion, dietary deprivation of DHA precursor β-LNA in the absence of dietary DHA reduced brain DHA and exacerbated NMDA induced AA and inflammatory markers in rat brain. Supplementation did not suppress upregulated NMDA induced markers in rat brain.
